# Arthroscopic-Assisted Acromioclavicular Joint Reconstruction

**DOI:** 10.7759/cureus.26036

**Published:** 2022-06-17

**Authors:** James R Satalich, Alexander Vap

**Affiliations:** 1 Orthopedic Surgery, Virginia Commonwealth University Hospital System, Richmond, USA; 2 Orthopedic Surgery, Virginia Commonwealth University, Richmond, USA

**Keywords:** shoulder scope, shoulder, arthroscopy, ac joint, hook plate

## Abstract

There are many documented techniques for the surgical treatment of a chronic acromioclavicular (AC) joint reconstruction, but unfortunately, there is no gold standard. Treatment options include hook plates, allograft reconstruction, and suture fixation, among many others.

This technique is an innovative method for looping the allograft around the coracoid and clavicle and using the hook plate for fixation. This avoids any drilling within the clavicle or coracoid, therefore decreasing the risk of fracture and ensuring the reduction of the AC joint.

## Introduction

Acromioclavicular (AC) joint injuries account for approximately 9% of all shoulder injuries and can lead to pain, scapular instability, and neurological symptoms, among others [1, 2]. When surgical intervention is recommended, the treatment options vary and all come with risks. Options include hook plate fixation, allograft reconstruction, and suture tape fixation, among others.

Known complications include loss of reduction, fracture, graft failure, and hardware failure, among others [3]. We present a novel technique in looping the graft around the clavicle and coracoid and secure it using a hook plate.

## Technical report

Diagnostic arthroscopy

The patient is placed in the beach chair position. A diagnostic arthroscopy is performed after making the standard posterior and anterior arthroscopic portals.

Exposing the coracoid

**Figure 1:**
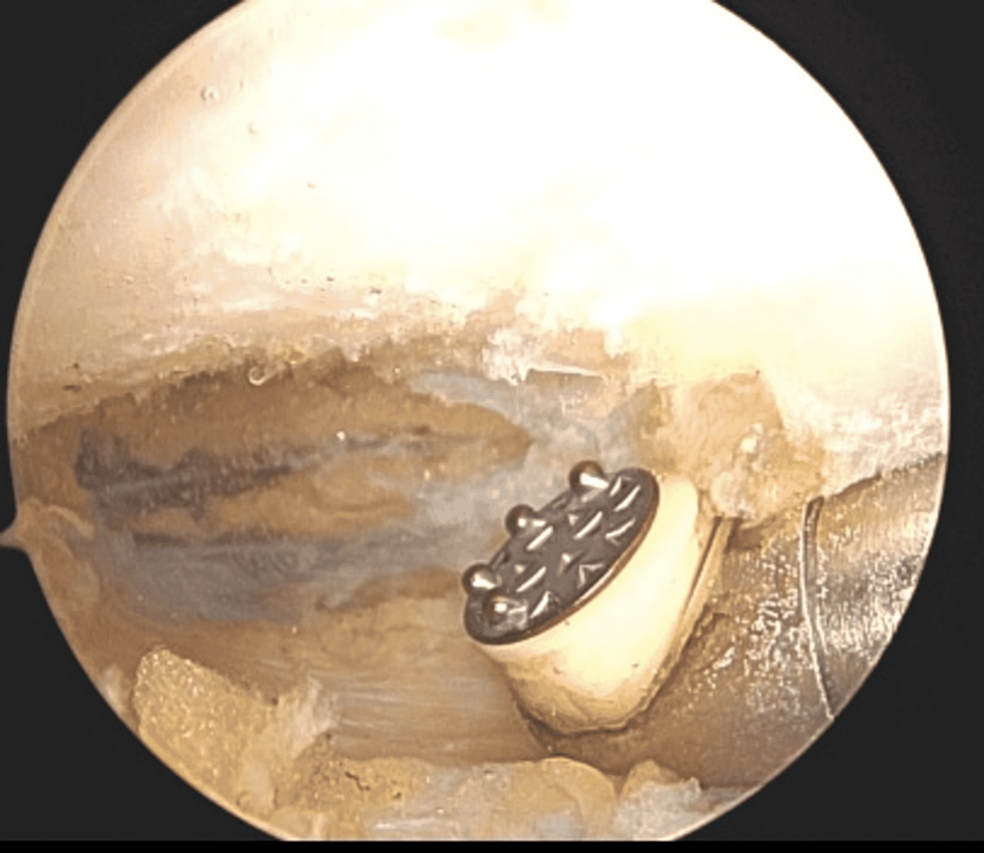
The coracoid is identified, and the surrounding tissue is cleaned by using the shaver and/or wand

Identify the coracoid and clean the underlying tissue and expose the bony surfaces (Figure 1). It’s critical to ensure the area under the coracoid is clean as the graft will be passed beneath it. After the coracoid is cleaned, make an incision over the distal clavicle and expose the AC joint.

Graft passage

**Figure 2:**
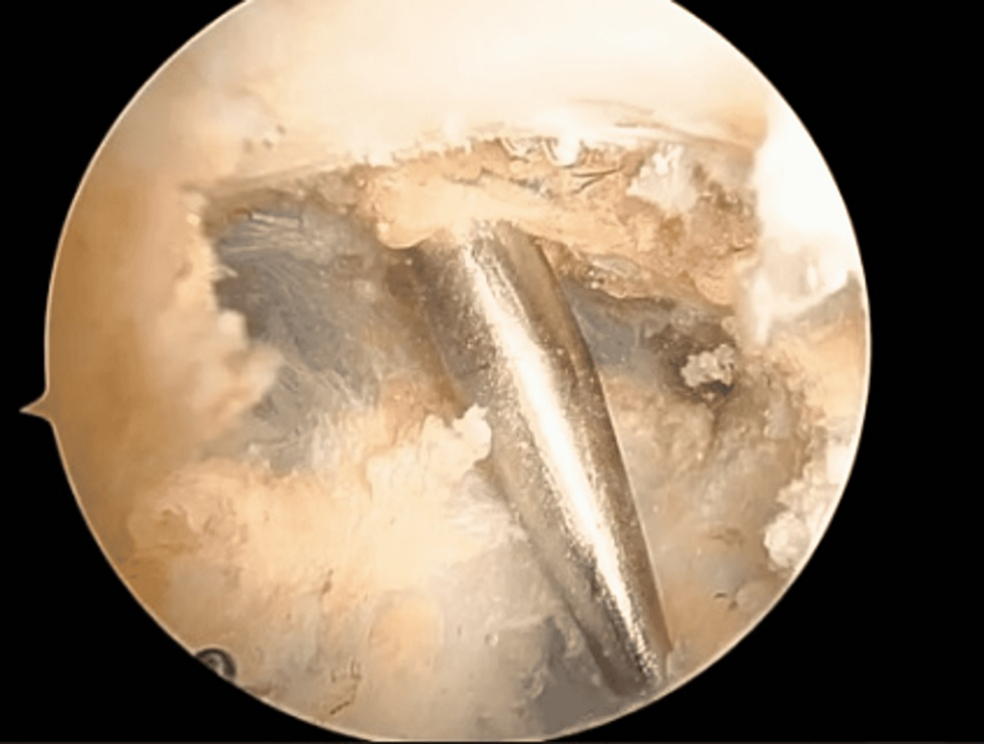
Place the switching stick posterior to the clavicle and medial to the coracoid.

**Figure 3:**
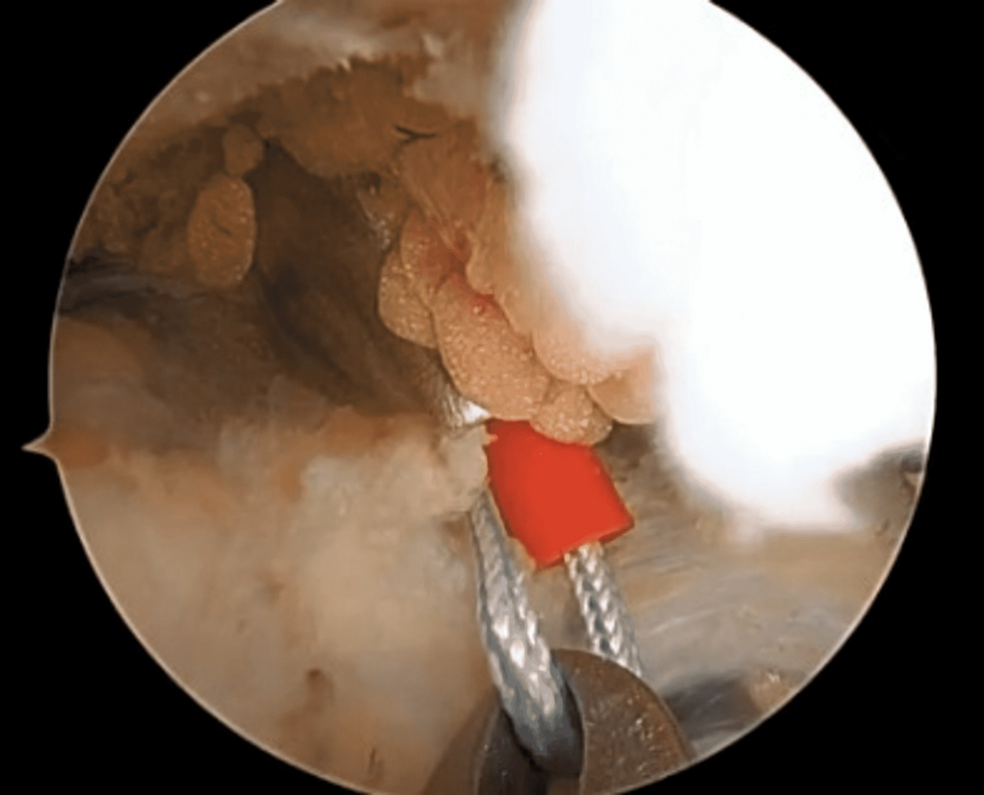
Insert the FiberStick suture into the joint.

**Figure 4:**
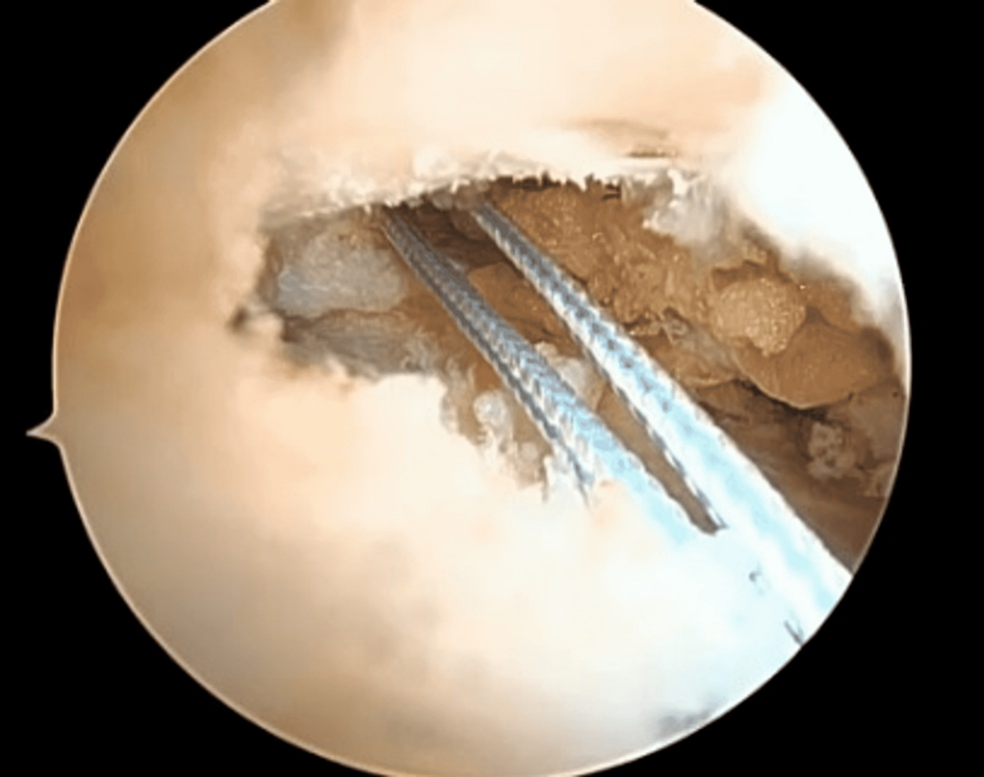
Shuttle the FiberStick suture through the accessory portal.

Use a switching stick, and place it posterior to the clavicle and medial to the coracoid. We ensure the scope is always visualizing the coracoid for stick placement. In obese patients, fluoroscopy can be used for assistance if it's difficult to identify bony landmarks. Use the arthroscopy to identify the switching stick and ensure it’s adjacent to the coracoid (Figure 2). Dilate over the switching stick. Place a FiberStick suture (Arthrex, Naples, FL) into the joint (Figure 3) and retrieve the suture (Figure 4). Standard anterior and posterior portals should be sufficient for visualization during this step.

**Figure 5:**
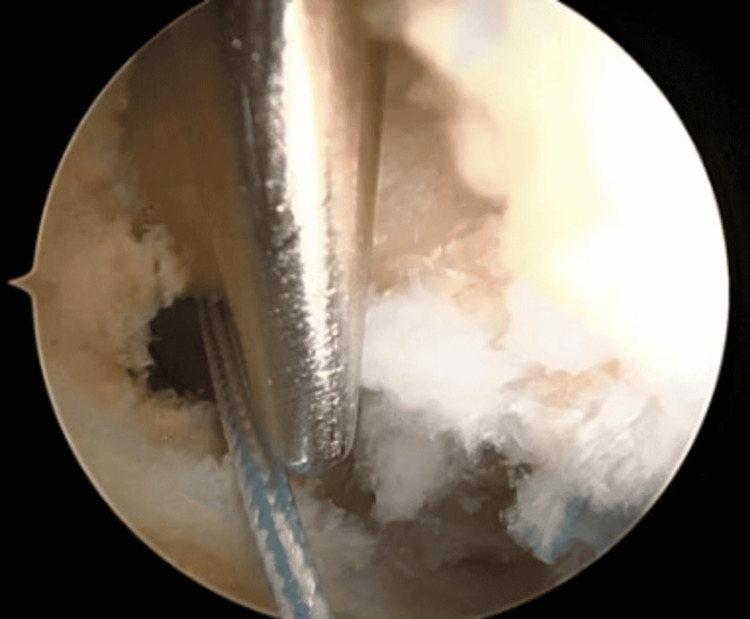
Insert the switching stick anterior to the clavicle and lateral to the coracoid.

**Figure 6:**
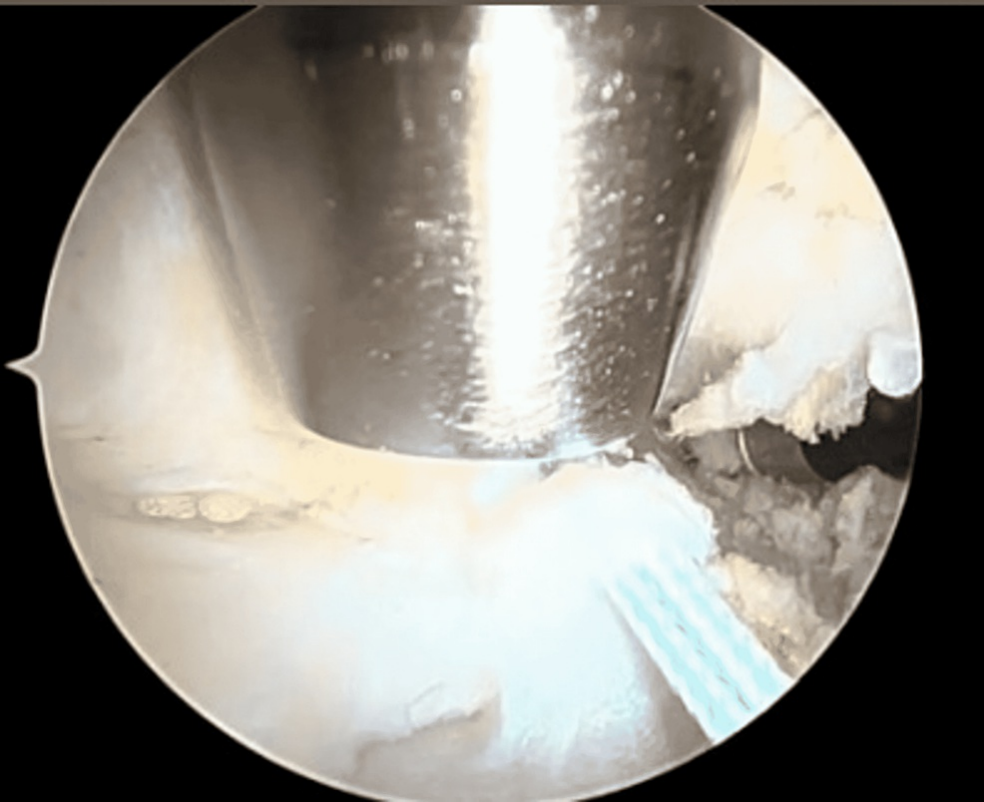
Shuttle the FiberStick suture through the accessory portal.

**Figure 7:**
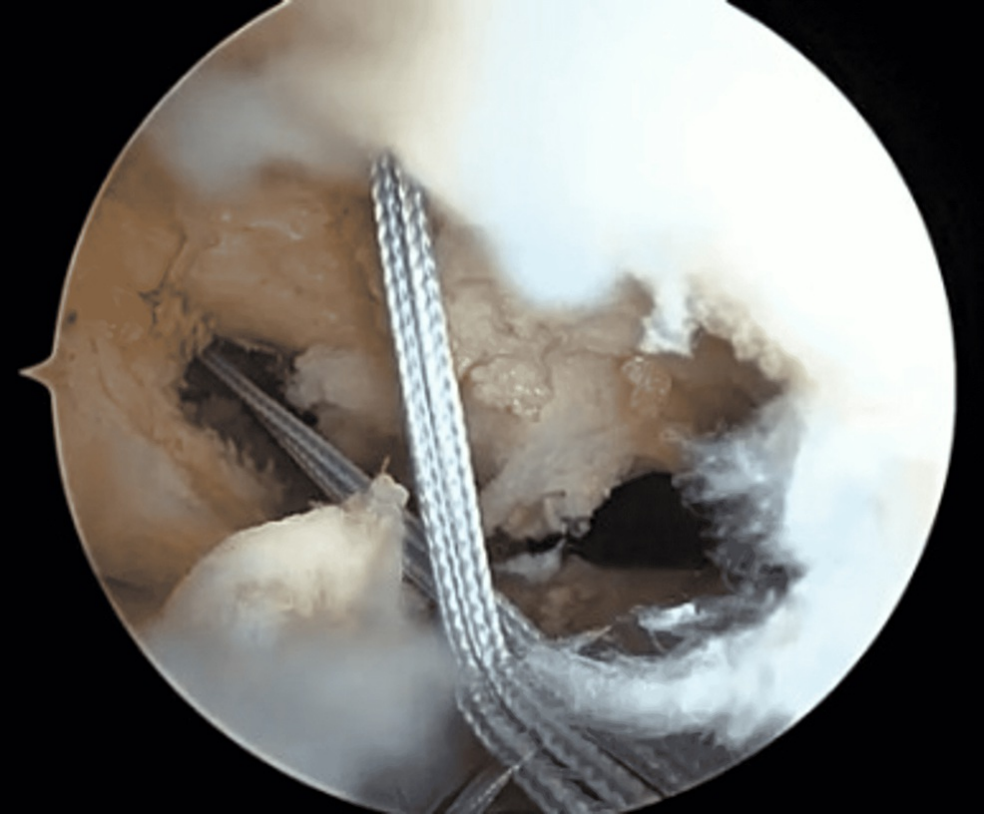
Both sutures are seen through the portal and ready for graft passage.

Next place the switching stick anterior to the clavicle and lateral to the coracoid (Figure 5). Dilate again (Figure 6). Under direct visualization using the scope, insert an additional FiberStick suture and pull out the suture (Figure 7).

**Figure 8:**
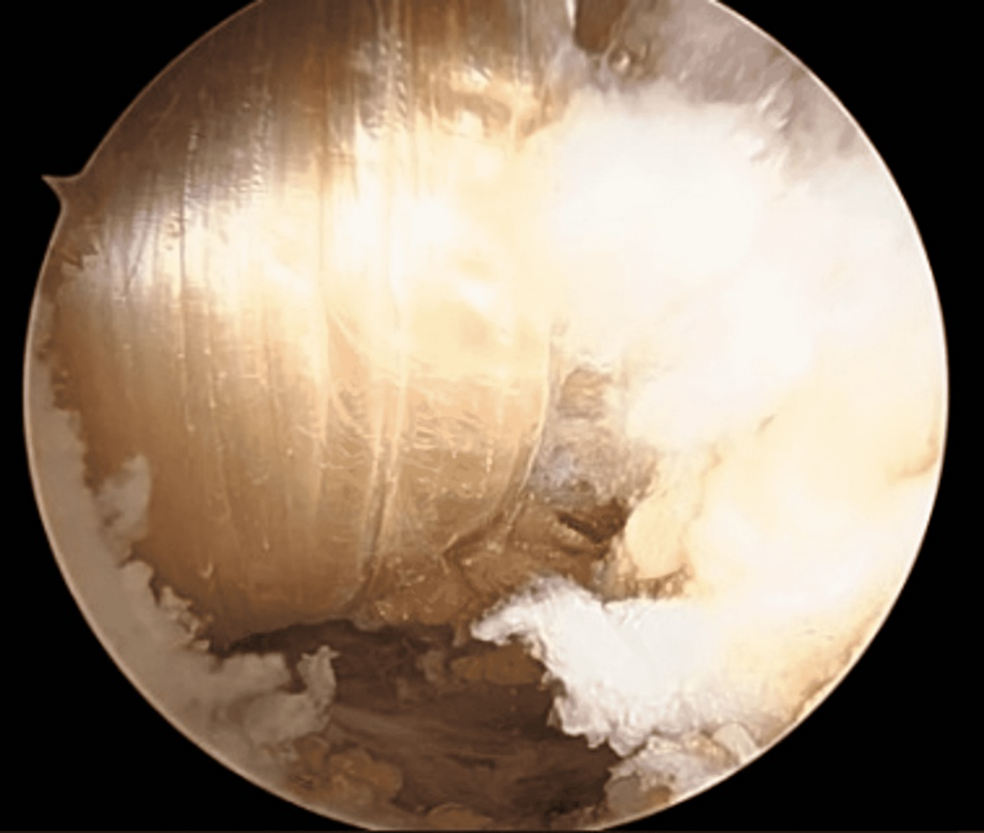
Pass the graft under the coracoid and on both anterior and posterior aspect of the clavicle, effectively creating a sling.

**Figure 9:**
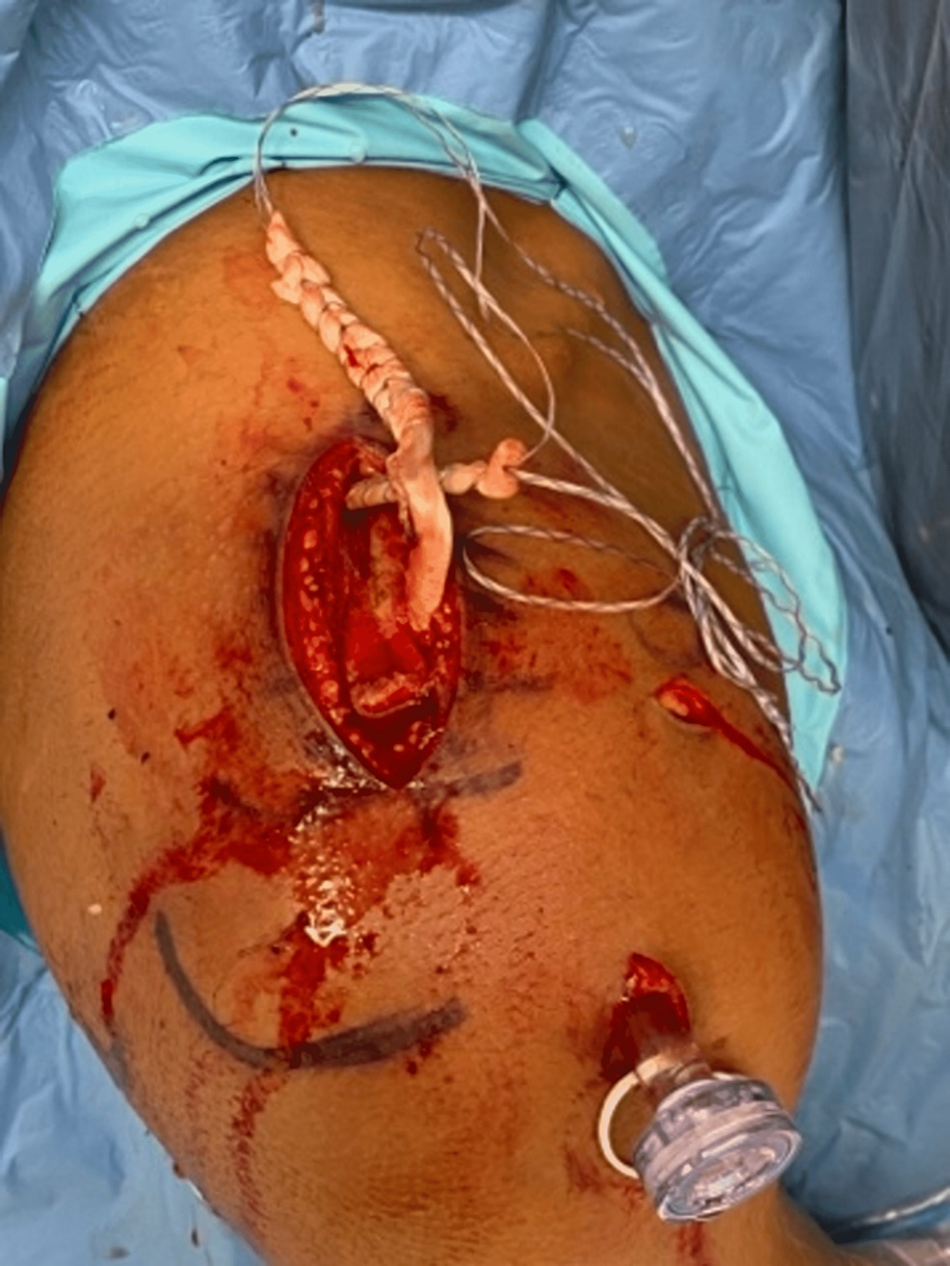
Both ends of the allograft are seen above the clavicle on opposite sides.

Using both sutures, shuttle the posterior tibialis allograft on both sides of the clavicle. Ensure it’s on the undersurface of the coracoid (Figure 8). The two tails of the graft are now on both sides of the clavicle, anterior and posterior, creating a lasso or sling under the coracoid (Figure 9).

Cross the two ends of the graft over the clavicle and tie it to itself using a #2 FiberWire (Arthrex, Naples, FL) in a figure-of-eight fashion. Again, using the scope, confirm the graft is secure under the coracoid after suturing it together.

Application of the hook plate

Using fluoroscopy, chose the appropriate size hook plate and place it over the graft. Confirm the AC joint is reduced on X-ray. Use standard technique to secure the hook plate down to the bone.

## Discussion

The AC joint is a common injury in today's population and can lead to pain, and scapular instability, among other symptoms if not surgically reduced and stabilized when indicated. There are various techniques for surgical reduction, including open versus arthroscopic, a combination of the two, and then the decision on fixation methods such as using hook plates, sutures, and buttons, among many others. Unfortunately, there is no gold standard technique. To avoid the risk of fracture and intra-op complications, we introduce this technique that includes arthroscopic assistance in looping the graft around the clavicle and coracoid to reduce and maintain the AC joint.

Coracoid fractures secondary to AC joint reconstructive reconstruction techniques still remain a complication [3]. Techniques that include intra-osseous buttons, screws, and suture tunnels have all been reported to cause intra-operative fractures [4, 5]. Milewski et al. reported up to a 20% prevalence of coracoid fractures caused secondary to drill holes used for passage of suture or the graft. Even popular techniques such as suture-button systems (Arthrex Dog Bone), require bony drilling which can increase the risk of coracoid fractures [6]. Biomechanical studies show that decreasing the hole size with the coracoid would decrease the risk of fracture [7]. Regardless of the size of the coracoid tunnel, it is reported in the literature that using a coracoid sling technique and avoiding bony tunnels result in fewer complications [8].

This technique has several advantages. This procedure is primary arthroscopic and avoids the large incision down to the coracoid for graft passage. The surgeon can use the scope to secure graft under the coracoid. Next, the hook plate not only secures the graft down to the clavicle but also acts as a mode to maintain the AC joint reduction. Lastly, as stated above, this technique avoids the risk of coracoid fracture secondary to drilling.

The main disadvantage to this procedure is the need to remove the hook plate. This typically occurs around six months post operatively and is typically indicated when the patient notes irritation from it. An additional disadvantage is the unknown fixation strength of suturing the graft tails.

Overall, to minimize the risk of coracoid fracture and large open incisions, we introduce a technique in which we loop the allograft around the coracoid and clavicle and secure it by suturing the tail ends. We avoid the additional drilling through the coracoid for the sole purpose of graft fixation. Further analysis is recommended for long-term outcomes using this technique.

## Conclusions

This technique provides surgeons with a technique for AC joint reconstruction that involves a combination of different techniques. This includes using the scope to loop the allograft around the clavicle and coracoid along with additional fixation using the hook plate to maintain the AC joint reduction. This technique effectively decreases the risk of coracoid fracture and large open incision to pass the graft. Further studies and long-term analysis should be evaluated.
